# A Rare, yet Classic Case of Colloid Cyst of Third Ventricle

**DOI:** 10.7759/cureus.16406

**Published:** 2021-07-15

**Authors:** Praveen BK, Adesh Shrivastava, Garima Goel, Hemlata Panwar, Neelkamal Kapoor

**Affiliations:** 1 Pathology and Laboratory Medicine, All India Institute of Medical Sciences, Bhopal, IND; 2 Neurosurgery, All India Institute of Medical Sciences, Bhopal, IND; 3 Pathology and Laboratory Medicine, AIl India Institute of Medical Sciences, Bhopal, IND

**Keywords:** third ventricular cyst, colloid cyst, obstructive hydrocephalus, benign ventricular cyst, sudden death

## Abstract

Colloid cyst of third ventricle is a rare, benign, congenital lesion that usually presents with headache, and associated with altered cognition, nausea, vomiting, gait ataxia, and blurred vision. A large cyst/growing cyst can cause obstructive hydrocephalus leading to acute rapid neurological deterioration and sudden death.

Here we report a classic clinical presentation and histopathological features of colloid cyst of third ventricle with specific emphasis on the importance of rapid diagnosis and management to avoid potentially fatal complications of this otherwise benign lesion. Newer modalities like neuroendoscopy or stereotactic aspiration of cyst are now the preferred choices of management.

Awareness of this entity for early diagnosis and management with minimally invasive procedures such as neuroendoscopy or stereotactic aspiration of cyst is crucial for better prognosis and patient care.

## Introduction

Colloid cysts of third ventricle are rare, benign, congenital lesions that comprise 0.5-2.0% of all intracranial lesions/tumors and 10-20% of all intraventricular lesions/tumors with an incidence of 3.2 cases per 100,000 people [1,2]. Large colloid cysts usually present with headache, and are associated with altered cognition, nausea, vomiting, gait ataxia, and blurred vision. Due to their particular location, a large cyst/growing cyst may cause obstructive hydrocephalus leading to acute rapid neurological deterioration and sudden death [3]. In about 10% of cases of colloid cysts of the third ventricle, sudden death has been reported. Of all cases of sudden death, 0.16-3.2% are caused by brain lesions, of which colloid cyst of the third ventricle is the main contributor [4]. Of all cases of sudden coma and death, these lesions contribute about 0.001-0.009% [4]. Due to this reason, identification of this lesion and its rapid management is needed, despite the benign nature and to prevent life-threatening complications.

## Case presentation

 A 24-year-old man presented with a short history of progressive headache, which was holocranial in nature with a feeling of heaviness, particularly aggravated in early morning since 15 days. This was associated with on and off type of vomiting, which did not subside with medication. He also complained of dizziness since five days. Patient was a known case of migraine, on treatment. There were no complaints of blurring of vision, seizures, or focal neurological deficits. Routine hematology and biochemical investigations were done and were normal.

**Figure 1:**
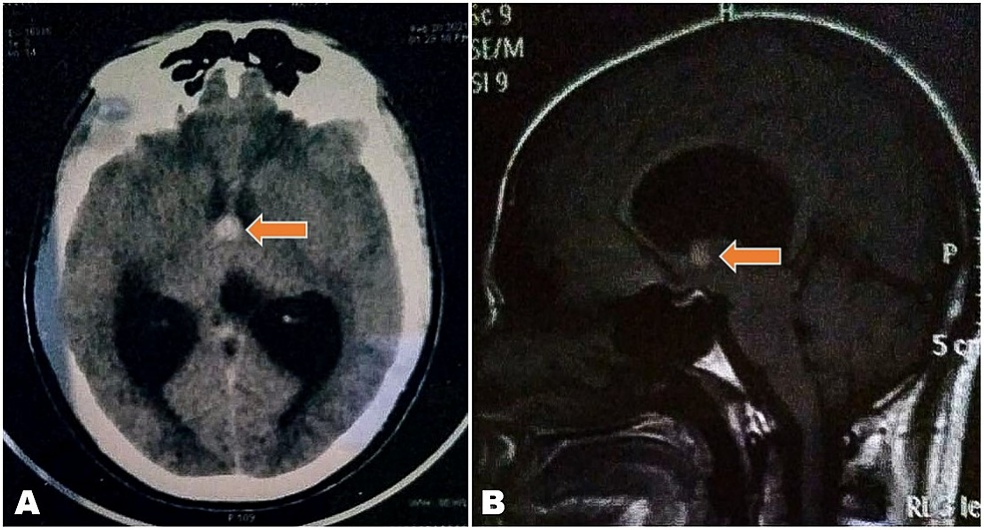
Brain CT Images A) Axial plain CT image showing dilation of bilateral lateral ventricles with a well-defined cyst in the region of the foramen of Monro with hyperdense contents within, suggestive of a colloid cyst. B) Sagittal T1-weighted non-contrast image shows a hyperintense well-defined cyst in the region of the roof of third ventricle causing obstructive hydrocephalus and effacement of the third ventricle.

Patient was immediately taken for further radiological investigation. Non-contrast CT brain was performed and it showed a well-defined rounded hyperdense cystic lesion measuring 1 x 1 cm in the roof of third ventricle associated with mild to moderately dilated bilateral lateral ventricle. There was no evidence of periventricular cerebrospinal fluid ooze or intraventricular hemorrhage (Figure 1A, 1B). Rest of the bilateral cerebral hemispheres were normal in attenuation. Brain stem and cerebellum revealed normal parenchymal attenuation. No midline shift was seen and bony calvaria appeared normal. Patient underwent endoscopic retrieval through right Paine's point. 

**Figure 2:**
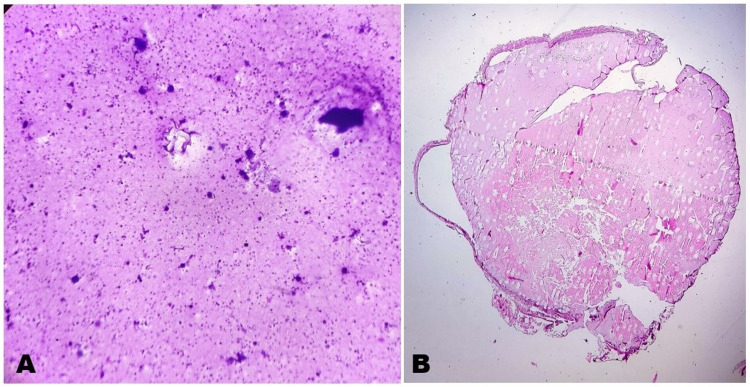
Cytology and HPE Whole-Slide Imaging A) Fluid cytology - Cytocentrifuge smears prepared at 1000 rpm for 10 minutes show thick amphophilic proteinaceous fluidic material in the background. There are scattered inflammatory cells consisting of histiocytes, lymphomononuclear cells, and cholesterol crystals (Giemsa stain, 10x). B) HPE section showing thin fibro-collagenous cyst wall, lined by pseudostratified-to-simple columnar epithelium (H&E stain, scanner view). H&E: hematoxylin and eosin; HPE: histopathological examination.

Intraoperatively cyst fluid was aspirated and was sent for fluid cytology. We received 0.5 mL slightly hazy fluid. The centrifuged smears examined show thick amphophilic proteinaceous fluidic material in the background. There were scattered inflammatory cells consisting of histiocytes, lymphomononuclear cells along with cholesterol crystals. Features were consistent with benign cystic lesion (Figure 2A).

**Figure 3:**
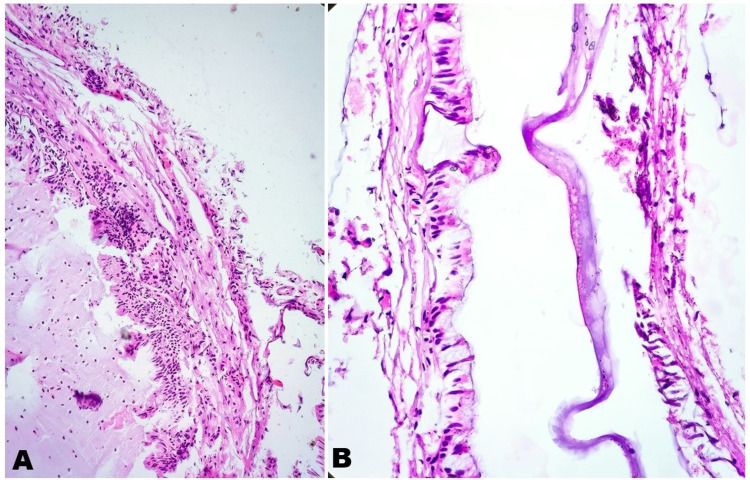
HPE Sections A) The cyst lumen shows abundant amorphous-to-granular eosinophilic material along with few macrophages, lymphocytes, and neutrophils (H&E, 10x). B) Thin fibro-collagenous cyst wall lined by pseudostratified-to-simple columnar epithelium having cilia ( H&E, 40x). HPE: histopathological examination; H&E: hematoxylin and eosin stain.

Histopathological examination showed a thin fibro-collagenous cyst wall lined by pseudostratified-to-simple columnar epithelium, focally ciliated (Figure 2B). The cyst lumen showed abundant, amorphous-to-granular eosinophilic material along with few macrophages, lymphocytes, and neutrophils (Figure 3A, 3B). Outer surface of the cyst showed few congested capillaries. Histopathological examination favored colloid cyst of third ventricle.

Postoperative period was uneventful. On close follow-up, patient was getting symptomatically better and no repeat imaging was required in this case.

## Discussion

Colloid cysts of third ventricle are rare lesions arising from ectopic endodermal migration in the velum interpositum or from the primitive neuroepithelium of the tela choroidea. These cysts are usually located in the rostral aspect of roof of third ventricle. They also project inferiorly to occur in the antero-superior quadrant adjacent to the foramina of Monro [1]. Colloid cysts are also rarely found in the pituitary gland and the fourth ventricle [5]. In the third ventricle they obstruct the foramina of Monro and cause acute hydrocephalus. These cysts noted to occur in the roof of third ventricle are pedunculus in nature, thereby causing intermittent obstruction and symptoms [6]. Presenting symptoms are varied, with the most common being episodes of paroxysms of headache (with postural variation), amblyopia, vomiting, and rarely mental changes. Other symptoms include but not limited to vertigo, disturbed mentation, drop attacks, sudden leg weakness, and rarely seizures. Recent studies even show the prevalence of cognitive symptoms ranging from anterograde amnesia to gustatory hallucination [1].

Sudden death has happened in about 10% of patients with colloid cyst of the third ventricle. Symptomatic colloid cyst of the third ventricle has the highest risk of acute deterioration in 34% cases with a mortality rate of 12% [4]. Persistent or intermittent obstruction of foramina of Monro can lead to acute lateral ventricle dilatation resulting in hydrocephalus intracranial hypertension, which can be fatal. Death may also be due to the compression of the hypothalamus by the cyst and subsequent cardiac reflex [7].

Both CT and MR imaging can be used in the diagnosis of colloid cysts. On CT scans, colloid cysts are slightly hyperdense compared to brain, but may occasionally be hypodense or isodense. Most colloid cysts are rounded to oval. Contrast-enhanced CT shows thin rim of enhancement and it represents the cyst capsule. Using MR imaging, colloid cysts usually have a variable appearance. MR imaging shows intracystic fluid levels or central and peripheral components in the lesion. Radiological differential diagnoses include small dermoid, craniopharyngioma, ependymoma, or an intraventricular glioma [6].

On histopathological examination, colloid cysts are lined by pseudostratiﬁed epithelium along with scattered ciliated cells, interspersed with mucous goblet cells. The cyst content is amorphous periodic acid-Schiff positive. Ultra-structurally ciliated cells and non-ciliated cells having microvilli, basal cells, goblet cells with secretory granules, and undifferentiated cells with scanty organelles may be seen. These ciliated cells are endodermal in origin but the topographical distribution of the ciliated epithelial cells suggests respiratory origin and distally tapering cilia suggests olfactory origin. Histopathological differential diagnosis includes ruptured epidermoid cyst, choroid plexus papilloma, or degenerated choroid plexus [8].

Some authors have suggested that cyst more than 1 cm should be surgically removed and neither the size, duration of symptoms, or ventricular dilatation seem to be reliable predictors. Microsurgical approaches like trans-callosal, trans-ventricular, and transcortical were traditionally the mainstay of treatment for colloid cysts. Newer modalities like neuro-endoscopy and stereotactic aspiration of cyst are minimally invasive procedures. Prognosis is excellent, if surgically managed on time [2].

## Conclusions

Thus, awareness of this entity for early diagnosis and management with minimally invasive procedures such as neuroendoscopy and stereotactic aspiration of cyst is crucial for better prognosis and patient care. Location, size, and direction of growth determine the prognosis of neglected cases. Preoperative and intraoperative diagnoses play a pivotal role. The entity of colloid cyst of third ventricle should always be considered in cases of acute headache, if other possibilities are ruled out. This also warrants further study to figure out the optimum timing, for surgical intervention in asymptomatic patients.
